# Annual Meeting of Governors and General Council

**Published:** 1914-05-09

**Authors:** 


### KING EDWARD'S HOSPITAL FUND FOR LONDON.

### Annual Meeting of Governors and General Council.

### Expenditure and Amalgamation Schemes in London.

The annual meeting of the Governors and General Council of King Edward's Hospital Fund for London, to receive the accounts and the report of the General Council for the year 1913, was held at St. James's Palace on April 30, his Highness the Duke of Teck being in the chair.

Those present were : The Viscount Iveagh; the Speaker of the House of Commons, the Right Hon. James W. Lowther, M.P.; the Chief Rabbi, the Rev. J. H. Hertz, D.D.; the Earl of Bessborough; Lord Sandhurst; Lord Rothschild; Lord Stamfordham; the Hon. H. L. W. Lawson, M.P.; the President of the Royal College of Physicians, Sir Thomas Barlow, Bart.; the President of the Royal College of Surgeons, Sir Rickman J. Godlee, Bart.; the Right Hon. Sir Savile Crossley, Bart., Honorary Secretary; the Right Hon. Thomas Burt, M.P.; the Right Hon. Sir Vezey Strong; Sir William S. Church, Bart.; Sir Owen Philipps; Sir William H. Bennett; Sir William J. Collins; Sir Cooper Perry; Sir John Craggs; Sir John Tweedy; Mr. Frederick M. Fry; Dr. Edwin Freshfield; Mr. John G. Griffiths, Honorary Secretary; Mr. Albert G. Sandeman; Mr. Algernon E. Sydney.

Investments of £2,000,000.

Lord Rothschild (the Honorary Treasurer), in presenting the accounts of receipts and expenditure and the balance sheet for the year ending December 31, 1913, said : It was his pleasant duty to present to the noblemen and gentlemen present the account of the receipts and expenditure for the past year. He did not propose to go into them, as members of the Council had them in their hands. But there were two matters that required some little explanation. As he had said at the meeting in December, the Fund had received a considerable sum of money from the Executors of the late Sir Julius Wernher, representing the first instalment of the one-twelfth share of the residue left to capital account, to the value of £270,000. As the result of the receipt of this instalment of the legacy, the total investments of the Fund now exceeded £2,000,000.

There was another legacy to which he ought to refer : that was, the Harman Bequest of ground-rents, worth £1,382 3s. a year. The surveyor's report, recently received, estimated the value of this most acceptable legacy at about £25,000.

His Highness the Duke of Teck formally moved the adoption of the accounts.

The resolution, having been seconded by Lord Iveagh, was then put and carried unanimously.

### The Council's Report.

Sir Savile Crossley (Honorary Secretary) presented the draft report of the Council for the year 1913, as follows :—

The total receipts for the year 1913 were £187,704 18s. This sum was made up as follows : Donations, £5,810 0s. 6d.; contributions to capital, £2,346 7s. 10d.; annual subscriptions, £26,187 17s. 9d.; contribution of the League of Mercy, £14,000; from the Lewis Estate, £9,092 4s. 3d.; legacies, £48,022 1s. 3d.; interest from investments, including property not yet transferred to the Fund, £82,046 6s. 5d.; together with £200 from the Trustees of the Bawden Fund.

The amount distributed was £157,500, being the same as in 1912. Of this sum the hospitals received £151,000, while the remaining £6,500 was distributed amongst consumption sanatoria and convalescent homes taking London patients.

The £151,000 allotted to the London hospitals was directed to be applied as follows : £109,350 to general purposes and maintenance; £6,000 towards the liquidation of debts on maintenance account; £1,500 to an amalgamation scheme; and £34,150 for additions and improvements to hospitals and their equipment. Of the £6,500 given to consumption sanatoria and convalescent homes, £3,800 was allotted in consideration of the allocation of beds for patients to be sent from certain London hospitals.

### Finance and Administration.

The total sum distributed amongst hospitals, convalescent homes, and consumption sanatoria during the last ten years (after deducting conditional grants to the extent of £7,409 16s. 7d. that have been allowed to lapse) is £1,357,840 3s. 5d.; since the foundation of the Fund a total amount of £1,790,916 8s. 5d. has been distributed.

During this year the amount spent on administration was £1 12s. 5d. per cent. of the total amount received, as compared with £1 17s. 1d. last year. The corresponding percentage for the whole period since the inauguration of the Fund has been £1 6s. 3d.

His Majesty the King, Patron of the Fund, has been graciously pleased to continue his annual subscription of £1,000. Her Majesty Queen Mary, her Majesty Queen Alexandra, his Royal Highness the Prince of Wales, and many other members of the Royal Family are also liberal supporters of the Fund.

The list of contributions includes annual subscriptions of £5,000 from Mr. Walter Morrison, £4,000 from the Drapers' Company, £1,000 from Mr. William Waldorf Astor, and £1,000 from the Merchant Taylors' Company; besides three anonymous donations each of £1,000.

A Member of the Council's Legacy.

The legacies received during the year included £25,000 from the late Sir Julius C. Wernher, Bart., £8,471 6s. 4d. on account of the bequest of the late Lord Lister, £6,000 from the late Mrs. Batchellor, £4,622 19s. 4d. from the late Sir William Dunn, Bart., and £1,000 from the late Mrs. Emma James.

In addition to the legacy of £25,000 from the late Sir Julius Wernher referred to above the Fund received in February of 1914 securities to the value of £269,644 7s. 2d., being a first instalment of his further legacy, left to the capital account of the Fund, of one-twelfth of the residue of his estate. This very important and generous gift is one of the largest ever received by the Fund, and is the more to be appreciated as it has been given by one who during his life assisted in the management of the Fund as a member of the Council.

Besides the legacies already mentioned, a bequest from the late Mr. A. H. Harman consisting of ground-rents amounting to £1,382 3s. per annum was in course of transfer to the Fund at the end of the year.

RECEIPTS FOR 1912 AND 1913 COMPARED.

The total amount received in donations and subscriptions as compared with 1912 showed, excluding contributions to capital, a decrease of £5,154. Legacies, however, showed an increase of £36,584 9s. 9d. as compared with 1912, or of £9,352 14s. 5d. as compared with the average of the five years 1908 to 1912.

The increase of legacies very nearly balanced the exceptional falling off in receipts from this source last year, and thus enabled the Council, in spite of the decrease in donations, not only to maintain its scale of distribution but also to replace the greater part of the amount drawn from reserves to make up the amount distributed in 1912. As only about one-half of the present annual distribution is provided for by the income from investments, the Fund is dependent for the maintenance of its awards upon the contributions received during the year.

The contribution from the League of Mercy this year was £14,000, being a decrease of £4,000 as compared with the sum contributed in 1912. The total amount received from the League of Mercy since its foundation in 1898 now amounts to £202,000, apart from the sums distributed by the League to institutions outside the area of the Fund's distribution. The Council desire to express their appreciation of this very substantial assistance derived from the efforts of a numerous band of voluntary workers.

### Hospital Expenditure.

The annual statistical report on the expenditure of London hospitals during 1912, issued in August 1913, showed that the cost of working at hospitals had increased since the previous year by about £22,000. The report points out that, in so far as this increase is due to the rise in prices and the continual introduction of more costly methods of diagnosis and treatment, it does not nullify the efforts towards economy which produced the previous savings, estimated at £47,000 a year. In order to assist the separate hospitals to ascertain whether their own expenditure was affected by special or avoidable causes, as well as by these general and unavoidable causes, additional tables were introduced into the report comparing the increases and decreases at the various hospitals with the average increase or decrease of each group.

OUT-PATIENT STATISTICS AND COST.

Various questions having arisen as to the application of the rules of the Revised Uniform System of Hospital Accounts dealing with out-patient statistics and cost, a Joint Committee of the King's Fund, the Sunday Fund, and the Saturday Fund was appointed at a conference held on the suggestion of the Sunday Fund to consider the subject. The Joint Committee called into consultation the committee of hospital secretaries which assisted in the preparation of the Revised Uniform System itself, and the three Funds finally issued a memorandum containing additional regulations and suggestions, designed to promote uniformity in the statistics of the various hospitals.

Every eligible hospital and consumption sanatorium which applied for a grant was inspected and reported upon by the Fund's Visitors, consisting as heretofore of one medical man and one layman in each case.

The scheme of the Convalescent Homes Committee for securing beds at consumption sanatoria and placing them at the disposal of London hospitals for the accommodation of patients urgently needing country treatment has again been extended, the number of beds secured for the coming year at five sanatoria being 44, as compared with 39 last year.

HOSPITAL ACCOMMODATION IN LONDON.

The Distribution Committee have carefully considered all schemes for the extension or reconstruction of hospitals which have been submitted in accordance with the request of the Council. The Fund has thus used its best endeavours to assist the hospital authorities in ensuring that when capital expenditure is undertaken adequate consideration is given to economy, efficiency, and the prospect of funds for future maintenance; and has particularly considered the relation of each scheme to the general question of hospital accommodation in London.

The General Council desire to express their sense of the loss the Fund has sustained by the death of Lord Strathcona, who was for many years a valued member of the Council, and who, by his munificent donation of £200,000 in 1902, contributed so greatly towards the establishment of the Fund on an assured financial basis. The Council also regret to record the deaths of Canon Barnett and Sir Trevor Lawrence, members of the Council, and of Mr. Alfred Willett, a former medical visitor and member of the Convalescent Homes Committee, and the Hon. Spencer Lyttelton, a visitor for many years. They also regret the retirement, which took effect at the end of the year, of Sir Ernest Cassel, Mr. J. Danvers Power, and Mr. J. Pierpont Morgan. Mr. Power's services as Honorary Secretary from 1904 to 1907 give him a special claim to the gratitude of all connected with the Fund.

The Council are indebted to Mr. Fry for having kindly undertaken the duties of Honorary Secretary, as colleague to Mr. Griffiths, during Sir Savile Crossley's absence abroad for part of the summer.

The President of the Local Government Board, in pursuance of his powers under the Act, has appointed Messrs. Deloitte, Plender, Griffiths and Co. to audit the accounts of the Fund for the year.

The Council are under great obligations to the press for the valuable services they have rendered in the past, and continue to rely upon their kindly co-operation in obtaining for the Fund wider publicity and support.

### The Duke of Teck's Speech.

His Highness the Duke of Teck, in moving the adoption of the report, said :

My Lords and Gentlemen,—It is not the custom of the Governors to make any lengthy speech at this meeting, but, before moving the adoption of the report, I will take the opportunity of referring to one or two matters.

I am sure we should all wish to express our very sincere thanks to the Finance Committee. Their task, now that the Fund's capital is so large, is one of great responsibility; and we are very deeply indebted to those who, in the interests of the Fund and the hospitals of London, undertake that responsibility and discharge it so well. We regret very much that, owing to his numerous other engagements, Sir Ernest Cassel is unable to continue his valued services on the Council. We shall always remember with gratitude his special donation of £31,500 to supplement by one-fifth the grants made by the Fund in 1911. I should also like to add, on behalf of the Governors and the Council, a word of thanks to Messrs. Barings, whose advice and assistance are always at the disposal of the Fund, and also to Mr. Leslie Vigers, to Mr. Leonard Messel, and last but not least to our Honorary Solicitors, Messrs. Freshfields, who have all in various ways rendered good service to the Fund in connection with the administration of its property.

Since we last met, in December, we have to deplore the loss, in the death of Lord Strathcona, of a distinguished member of the Council and a great benefactor to the Fund. It is hardly too much to say that his generous donation of £200,000, received in 1902, together with a similar amount from Lord Mountstephen, laid the foundations of the capital fund of the Corporation which now forms such a valuable element in its stability. It would be impossible to exaggerate the importance of these gifts in the history of the Fund. But they were not the only sign of Lord Strathcona's interest in its work, and it is with feelings of sincere sorrow that we miss from our meetings the presence and assistance of a friend to whom we owe so much.

### The King's Message.

I have now the honour to read the following letter, addressed to the Hon. Secretaries by Lord Stamfordham :

"Buckingham Palace,

"April 29, 1914.

"Dear Sir,—I am commanded by the King to ask that the following message may be delivered at the Council meeting of the Fund to be held at St. James's Palace to-morrow :

"'While deeply deploring the loss sustained by the death of one of our great benefactors, Lord Strathcona, and of other friends, we must all be proud of the continued growth of the Fund and of the proof of confidence in its management shown by the most munificent bequest of Sir Julius Wernher, which will be of the greatest service. His splendid example will long be remembered by all interested in hospital work.'

"Yours very faithfully,

"Stamfordham."

It was also with great regret that we learned before the end of the year of the death of Sir Trevor Lawrence, whose resignation was announced at the last Council meeting. He had done the Fund good service for many years, both on the Council and on the Distribution Committee.

I also wish at this time to pay a tribute of gratitude, on behalf of the Governors and the Council, to the memory of Sir Julius Wernher, to whose munificent bequest of one-twelfth share of the residue of his estate the capital account of the Fund is indebted for another large increase. Since the end of the financial year we have received, as a first instalment of this legacy, securities to the value of £270,000. The resulting very valuable increase in the permanent income of the Fund will do much to ensure our annual distribution against the danger of possible reduction which seemed a year ago to be threatening. Sir Julius Wernher had served the Fund as a member of the Council ever since its foundation in 1897, and we receive this great gift with gratitude, not only as a welcome addition to the funds which we administer, but also as a token of the confidence which he felt in the management of the Fund, and as evidence of the strong appeal which the cause of the hospitals made to the administrative ability and generous impulses of one who had made himself acquainted with their work and with their needs.

I now beg to move the adoption of the report.

The Speaker of the House of Commons seconded the motion, which was carried unanimously.

The Report on the Throat Hospitals Amalgamation.

Sir William Church (Chairman of the Distribution Committee) moved the adoption of a report of the Distribution Committee upon the Throat Hospitals Amalgamation as follows :—

The Distribution Committee beg to report that the Hospital for Diseases of the Throat, Golden Square, and the London Throat Hospital, Great Portland Street, have entered into an agreement for the amalgamation of the two hospitals upon the site of the Golden Square Hospital and an adjoining site recently acquired by that hospital.

In 1907 the Fund offered, towards a general scheme for the amalgamation of the throat hospitals, a capital grant of £10,000 and an annual maintenance grant of £2,000 a year for five years. In addition to this, grants amounting in all to £10,500 were set aside between 1906 and 1911 out of the annual distribution for the purpose of a general amalgamation in lieu of maintenance grants to the separate institutions. In 1912 the Council decided that the £10,500 set aside should be added to the £10,000 promised, and that the whole £20,500 should be considered as a grant to capital including building capital.

### The Distribution Committee's Recommendations.

The Distribution Committee recommend that, out of the grants so promised and set aside for a general amalgamation, the following grants be made and promised towards the present partial scheme—namely, a capital grant of £10,000 and an annual maintenance grant (subject to the usual annual inspection) of £900 a year for five years.

The Distribution Committee further recommend that power be given to them to draw upon the balance of the capital and maintenance grants so promised or set aside, in such proportions as they may consider appropriate, in the event of any further amalgamation scheme being brought to a satisfactory conclusion before the next distribution.

The motion, having been seconded by Mr. Frederick M. Fry, was put and carried unanimously.

In the absence of Mr. Latham, K.C. (Chairman of the Convalescent Homes Committee), Dr. Freshfield moved the adoption of a report of the Committee upon the question of the reserved grant to the Maitland Sanatorium.

The adoption of the report was seconded by Sir William Bennett and carried unanimously.

Lord Sandhurst moved a vote of thanks to his Highness the Duke of Teck for presiding and for his conduct in the chair.

The Hon. Harry Lawson, M.P., in seconding the resolution, said his Highness had made himself a great name in the world of charity; and he was untiring in his zeal and in his good work on behalf of this Fund.

The resolution was carried unanimously.

The Duke of Teck having briefly acknowledged the vote of thanks, the proceedings terminated.

